# Where Does Bluetongue Virus Sleep in the Winter?

**DOI:** 10.1371/journal.pbio.0060210

**Published:** 2008-08-26

**Authors:** Anthony Wilson, Karin Darpel, Philip Scott Mellor

## Abstract

Bluetongue recently spread to northern Europe for the first time. Outbreaks in temperate regions are often interrupted by cold weather, but may reappear months later. Where, then, might bluetongue virus sleep in the winter?

Bluetongue virus (BTV) is spread by the bites of Culicoides midges (Figure 1), and can infect ruminant livestock such as cattle, sheep, and goats, wild ruminants such as deer, and camelids. Some infected animals develop the disease known as bluetongue, with clinical signs ranging from apathy and weight loss to swollen heads, tender feet and death (Figure 2). Historically a tropical and subtropical disease, bluetongue has become a regular visitor to southern Europe in the last decade [1,2]. Although growth in the global trade in livestock may have increased the frequency with which exotic viruses are introduced into Europe, the increasing tendency of those introduced strains to persist and spread is probably best explained by changes to the European climate [3], and several direct and indirect links between climate and BTV transmission have been identified [2]. BTV reached northern Europe for the first time in 2006, and affected around 2,000 holdings before reports ceased in early January 2007. The outbreak then re-emerged months later [4] and spread to a further 45,000 holdings by the end of the year, making it the most economically damaging outbreak of bluetongue ever seen [2,5].

**Figure 1:**
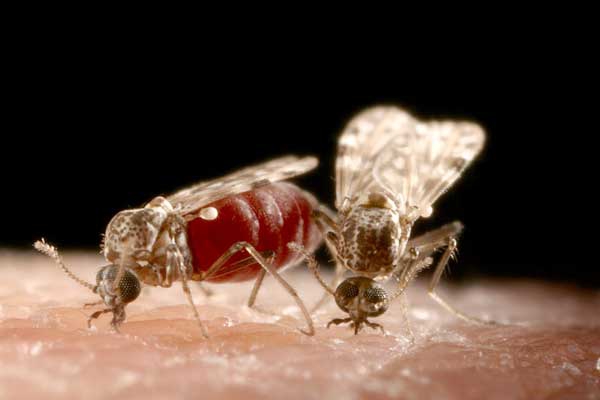
Blood-Feeding Culicoides Midges

**Figure 2:**
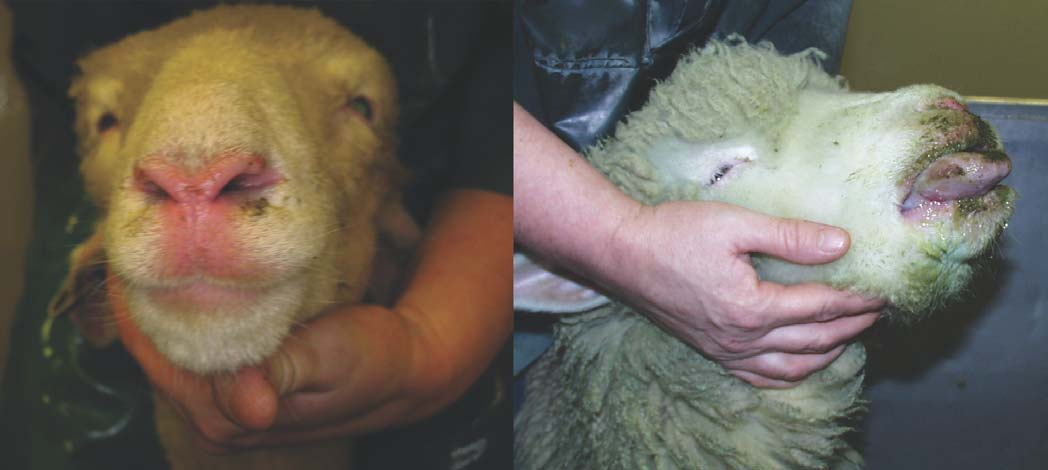
Moderate (Left) and Severe (Right) Clinical Signs of Bluetongue in Sheep

Although this re-emergence was a timely reminder of the ability of BTV to escape detection for months at a time in temperate regions, the ability of BTV to “overwinter” has been recognised for decades (e.g., [6–8]). Both midge activity and virus replication in the midge cease at cool temperatures, interrupting transmission, but outbreaks sometimes resume after “silent” periods of several months—far longer than the typical lifespan of the adult vector or the normal period of infectious viraemia in a mammalian host [7–9]. Where, then, might the virus persist without detection during the winter months?

## Where to Start? Lessons from Other Insect-Borne Diseases

**Figure 3:**
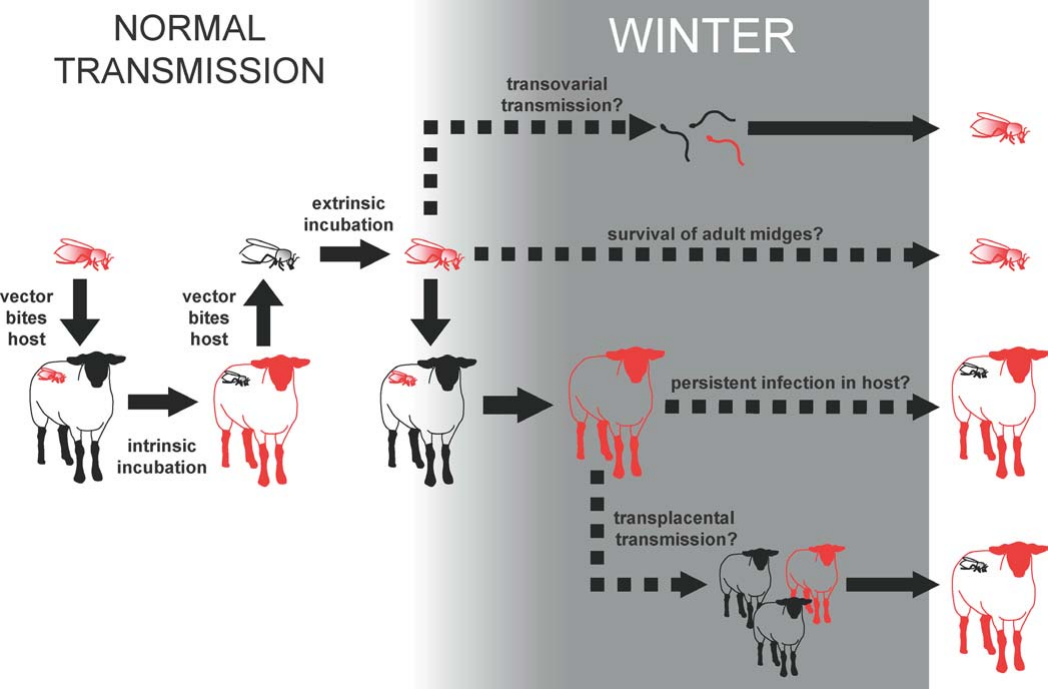
The Transmission of Bluetongue Virus in Summer (Left) and Winter (Right) Mechanical transmission, sexual transmission, and alternative vector and host species not shown.

The normal transmission cycle of a vector-borne disease such as bluetongue is shown in the left panel of Figure 3. Infectious vectors inject virus into hosts via saliva while feeding. Infected hosts then pass through a short incubation period before developing an infectious level of virus, and vectors that bite the host while virus levels are high may be infected. Ingested virus replicates in infected vectors until it reaches the salivary glands, at which point the vector becomes infectious and the cycle is completed. This same basic cycle of transmission is seen in vector-borne diseases from chikungunya to tick-borne encephalitis.

When contact between the primary vector population and primary host population is interrupted, there are three ways that a virus could theoretically persist: (1) in the vector population, (2) in the host population, or (3) via an alternative transmission cycle involving one or more novel vector or host populations. Persistence in the vector or host populations may be achieved via horizontal (direct) transmission between individuals, vertical transmission from infected parent to young, or persistence in individuals. Because insect vectors are generally infectious for life but relatively short-lived, persistence in individual vectors would require the survival of infected vectors for unusually long periods.

Examples of many of these potential routes have been seen in other vector-borne disease systems. Culex pipiens, an important vector of West Nile virus (WNV), survives the winter as overwintering adults that can tolerate temperatures as low as −25 °C (P. Reiter, personal communication), and that may be infected by WNV [10]. Vertical transmission in the vector population has been detected in several viruses, including Ross River virus [11] and WNV [12], and WNV may also be sexually transmitted between vectors [13]. Meanwhile, a number of arbovirus infections appear to be able to cross the mammalian placenta, including Getah virus, Ross River virus, and Western equine encephalitis [14,15], and birds that appear to have recovered from WNV may still harbour infectious virus in internal organs such as the spleen [13].

## Overwintering in the Insect Vector

Because most species of Culicoides at northern latitudes survive the winter as larvae [16], it might appear that the most likely mechanism for overwintering is the vertical transmission of virus from infected vectors to offspring via the developing egg (“transovarial” transmission; see Figure 3). However, experiments designed to look for vertical transmission of BTV in Culicoides have consistently reported negative results (reviewed in [17]). One field study detected fragments of BTV RNA in larvae but no live virus [18], supporting the hypothesis that pores in the vitelline membrane sometimes permit proteins, RNA fragments, and other materials less than 11 nm in diameter to enter the developing egg, while mechanically preventing the passage of intact virus particles, which are much larger [19].

**Figure 4:**
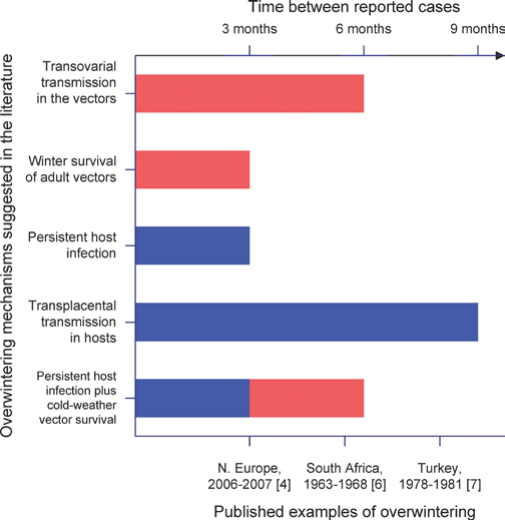
The Ability of Various Mechanisms to Explain Observed Instances of Overwintering

Might BTV be able to survive the winter in the adult vector population, then, as shown for West Nile virus? Although adult Culicoides are far less tolerant of sub-zero temperatures than Culex mosquitoes and are normally thought to survive no longer than 10–20 days [20], laboratory studies suggest that this lifespan may be extended by mild winter conditions, with individuals surviving for up to three months at 10 °C in the laboratory [21]. The winter of 2006–2007 was the mildest on record in Europe (J. Kennedy, personal communication), and small numbers of adult Culicoides vectors were caught throughout the winter period [22]. In mild winters such as this, it is possible that a small fraction of the infected adult Culicoides population might survive long enough to bridge the gap between transmission seasons (Figures 3 and 4). Adult Culicoides may also be sheltered from the worst conditions of winter to some degree by their choice of resting place. Although most north European Culicoides species were previously believed to be reluctant to enter buildings, studies during the current outbreak have suggested that small numbers may move indoors when outdoor temperatures begin to drop [23].

## Overwintering in the Ruminant Population

BTV could also covertly persist in the ruminant population during the winter, whether by chronic or latent infection of some individuals, transmission across the placenta from mother to foetus, or horizontal transmission during sexual intercourse. Chronically infected hosts remain continually infectious for prolonged periods of time, while latent viruses such as herpes lie dormant within an infected host but can be stimulated to resume replication by certain events, such as when immunity is compromised due to stress or other infections or following the use of certain pharmaceuticals such as steroids. Because latent infections are characterised by the absence of detectable virus, this type of persistence causes the greatest headache for surveillance and control. However, since a chronic infection may involve only single organs, it is not as easily distinguishable from a truly latent infection as it might first appear; BTV and most other arboviruses are normally detected by testing blood samples, and virus infecting only certain organs of the body but absent from the bloodstream may escape detection.

There is some evidence to support the idea of chronic BTV infections in ruminants. Infectious BTV can be isolated from the blood of cattle for much longer than from sheep and goats, and although the vast majority of infections in cattle endure for less than 60 days, a fraction may last for much longer [24]. Such infections could permit the virus to persist for three to four months without infecting new hosts, and thereby survive short periods of vector absence (Figures 3 and 4). Evidence for latent infection, meanwhile, was first reported over 30 years ago, when it was observed that feeding large numbers of uninfected vector Culicoides on apparently recovered cattle could stimulate the recrudescence of the virus [25]. Though this experiment was later criticised due to flaws in the research protocol, a more recent study confirmed that it was possible to isolate BTV from the skin of apparently recovered sheep over two months post-infection following Culicoides feeding [26]. Although other studies have not yet replicated this result, it would provide an elegant and efficient mechanism for virus to reappear in exactly the correct place and time to re-infect the next vector generation.

In transplacental infections, virus infecting the mother crosses the chorion and invades the foetus. Since the gestation period of cattle is nine months (against five for sheep), the potential for the virus to bridge the seasonally vector-free period via this mechanism is clear (Figures 3 and 4). Transplacental transmission of BTV was first documented in the 1970s [27], and suggested that the outcome was very much dependent on the stage of pregnancy at which infection occurred. Infection in early pregnancy was likely to result in abortion or stillbirth, while foetuses infected at a late stage of pregnancy tended to clear the virus. Infection at an intermediate stage, before the foetus's immune system is fully developed, sometimes resulted in a prolonged infection, and some lambs infected in utero remained viraemic for up to two months after birth [27]. However, the virus strains used in these experiments had been passaged in artificial tissue culture systems, and the relevance of these findings to the transmission of field strains of BTV was questioned. Recent reports from northern Europe appear to indicate that the strain of BTV currently circulating has the potential to spread vertically in the ruminant population [28,29,30], although the fact that it has never been detected previously implies that only a subset of field strains may have this capacity.

Infected bulls and rams may also occasionally shed the virus in semen, possibly because of contaminating blood cells, and venereal transmission may thereby occur. However, all research suggests that this occurs only during the normal duration of viraemia, and current trade regulations require that breeding bulls and rams are tested for BTV before semen can be exported.

## Other Possibilities

Various other overwintering mechanisms have been proposed, although generally with less supporting evidence than the mechanisms already described. For example, it has been suggested that BTV might be maintained in an as yet unknown reservoir host population with a long viraemia in which clinical signs either do not occur or go unnoticed. Such a reservoir host would need to demonstrate a duration of viraemia matching or exceeding that seen in cattle to account for most observed instances of overwintering, and although the duration of BTV viraemia in European wild ruminants has not been well studied, white-tailed deer and elk appear to show a detectable viraemia for only 16 days or less [31,32]. However, one study has suggested that viraemia in elk may resume up to three months post-infection in response to stress [31]. Coupled with the observation of relatively high levels of seroprevalence in red deer populations in Belgium during the current outbreak [33], this hypothesis may therefore warrant further investigation.

Other authors have suggested that, while BTV transmission is reduced by cold conditions, “overwintering” is effectively a consequence of intermittent surveillance, and that transmission within the recognised domestic ruminant hosts continues at low levels throughout the winter. Although most BTV cases are subclinical, and therefore a small number of infections might escape detection, this proposal has not received wide acceptance, and the occurrence of overwintering in regions such as northern Europe (where outdoor temperatures during the winter are indisputably below the requirements of the virus for replication in the vector) makes it even less likely. However, cold weather could, directly or indirectly, trigger behavioural changes in vector populations. Before malaria was eradicated from northern Europe, the winter stabling of livestock encouraged normally exophilic mosquito vectors to venture indoors, resulting in seasonal indoor malaria transmission [34]. Similarly, if adult Culicoides do enter buildings in the winter, they might experience conditions that are sufficiently warm for blood-feeding all year round.

Vectors other than Culicoides could also have a role to play. Even in cold weather, the Culicoides lifespan is relatively short, but ticks may also occasionally act as biological vectors of BTV [35] and can live for several years. Furthermore, mechanical transmission of BTV has been shown in Melophagus ovinus, a wingless ectoparasitic fly that lives in the fleece of sheep [36], and may be possible by other species. Mechanical transmission is the physical transportation of infectious virus from one host to another, without extrinsic replication. Because extrinsic virus replication is not required for this type of transmission, the minimum temperature threshold for the completion of the transmission cycle is removed and transmission can occur at any time of year irrespective of climate.

Finally, oral transmission between mammalian hosts may also be a possibility. Cows will readily consume placental material following a birth, and although transmission between ruminants in this way has not been confirmed, at least one possible example has been reported [28]. Carnivores can also become infected after ingesting BTV-infected meat and organs [37]. BTV can also be spread mechanically between animals by humans, for example through contamination of vaccines [38], but this should not occur if high production and hygiene standards are adhered to.

## Towards an Explanation

BTV is not limited to the core vector-borne disease transmission cycle commonly presented in textbooks. Multiple vectors and hosts may be involved, along with such diverse transmission routes as transplacental, mechanical, and oral routes. Rather than a single “missing link” enabling the virus to persist during the winter period, experiments have revealed a toolbox of possible mechanisms, with the potential to interact with and complement one another. For example, chronic infection of the uterus could increase the likelihood of transplacental transmission, which in turn may create chronically or latently infected calves. Examples of BTV overwintering in the literature can be found ranging from as little as three months to eight or nine. The shortest of these periods could be bridged by any of several candidate mechanisms, and the principal difficulty faced by the epidemiologist is narrowing down the list of suspects. At the other end of the scale, only a subset of these mechanisms or the interaction of two or more in sequence would be able to account for longer overwintering periods (Figure 4).

Although several of the mechanisms discussed in this paper were first suggested decades ago, interest in confirmatory and quantitative studies remained low for two reasons. Firstly, prior to the advent of molecular techniques, the subclinical nature of most BTV infections meant that it was both difficult and expensive to assert that transmission had stopped, and then to demonstrate that the re-emergence of bluetongue in a region was due to overwintering rather than reintroduction. Secondly, endemic regions such as South Africa or the United States have lived with bluetongue for so long that their agricultural sectors have adapted to its presence, avoiding the most susceptible breeds, vaccinating valuable animals, and accepting small losses rather than embarking upon expensive eradication programmes. Mechanisms that are important only for persistence therefore received little attention.

The recent spread of bluetongue into areas such as northern Europe, which are both highly susceptible to the economic effects of the disease and cold enough that classical transmission is interrupted for a significant part of the year, has stimulated field, laboratory, and analytical research into the mechanisms by which bluetongue may overwinter in temperate climes. The next steps for bluetongue research must be first, to establish these newly discovered transmission routes beyond reasonable doubt and second, to determine their importance for the transmission of BTV in the field. Some of this work has already begun: for example, several countries in northern Europe are conducting field studies to determine how readily their indigenous vector Culicoides enter animal housing [39], and the Institute for Animal Health, the Veterinary Laboratories Agency, and Animal Health are currently collaborating on a project to determine the degree to which transplacental transmission of BTV might be occurring in the current outbreak. Previous studies of similar diseases may also suggest productive approaches. For instance, direct surveillance for inactive adult vectors during the winter confirmed that they represented a potential mechanism for the overwintering of WNV [10], and similar surveillance of Culicoides populations, possibly via suction sampling, might shed further light on the potential for this route to maintain BTV during the winter months. Such detective work is not purely of interest for understanding the current outbreak in northern Europe. Confirmation of transplacental transmission of BTV in the field, for example, would have drastic consequences for international trade.

However, the epidemiology of bluetongue and other vector-borne diseases is determined by a number of factors, ranging from molecular variation in virus strains, through regional farm management and animal husbandry approaches, to spatial and temporal patterns of change in land use or climate detectable only via detailed analysis of high-resolution climate data or remotely sensed imagery. To achieve a full understanding of the epidemiology of diseases such as bluetongue will therefore require not only short-term independent entomological or veterinary studies but a commitment to truly interdisciplinary programmes of study over longer periods, incorporating approaches ranging in scale from molecular biology and virology through entomology and veterinary science to land use and climate modelling. Perhaps most importantly, the fact that we are now having to re-examine so many basic questions about the behaviour of Culicoides illustrates that we neglect basic studies of the ecology of insect species capable of acting as vectors of disease at our peril.

The existence of so many potential overwintering mechanisms for viruses such as BTV and WNV may also teach us more general lessons about the capacity for other medically or epidemiologically important vector-borne diseases to survive in temperate zones with seasonal vector absence. In particular, the risk to northern Europe from the BTV-related epizootic haemorrhagic disease virus (which also affects ruminants) and African horse sickness virus (which affects equids) should be re-evaluated. Consideration of the routes reviewed here may also be useful when assessing the risk of re-emergence of outbreaks of insect-borne viruses not previously seen in Europe, such as the 2007 outbreak of chikungunya virus in Italy. Meanwhile, as long as our understanding of overwintering in the field remains poor, the reappearance of BTV in northern Europe this year seems both inevitable and imminent.

## Abbreviations

BTV: bluetongue virus
WNV: West Nile virus

## Note Added in Proof

Since this manuscript was submitted, new cases of bluetongue have been confirmed from northern France (S Zientara, personal communication), Belgium [40], and Germany [41], indicating that BTV has once again overwintered in northern Europe.

## References

[pbio-0060210-b001] Mellor PS, Carpenter S, Harrup L, Baylis M, Wilson A, Mellor PS, Baylis M, Mertens PPC (2008). Bluetongue in Europe and the Mediterranean basin. Bluetongue.

[pbio-0060210-b002] Wilson A, Mellor P (2008). Bluetongue in Europe: Vectors, epidemiology and climate change. Parasitol Res.

[pbio-0060210-b003] Purse BV, Mellor PS, Rogers DJ, Samuel AR, Mertens PPC (2005). Climate change and the recent emergence of bluetongue in Europe. Nat Rev Microbiol.

[pbio-0060210-b004] International Society for Infectious Diseases (2007 June 13). Bluetongue—Europe (14): BTV-8, Germany (Nordrhein-Westfalen), confirmed. http://www.promedmail.org/pls/otn/f?p=2400:1001:2689187839676304::::F2400_P1001_BACK_PAGE,F2400_P1001_ARCHIVE_NUMBER,F2400_P1001_USE_ARCHIVE:1001,20070613.1928,Y.

[pbio-0060210-b005] Hoogendam K (2007). International study on the economic consequences of outbreaks of bluetongue serotype 8 in north-western Europe.

[pbio-0060210-b006] Nevill EM (1971). Cattle and Culicoides biting midges as possible overwintering hosts of bluetongue virus. Onderstepoort J Vet Res.

[pbio-0060210-b007] Taylor WP, Mellor PS (1994). Distribution of bluetongue virus in Turkey, 1978–81. Epidemiol Infect.

[pbio-0060210-b008] Osmani A, Murati B, Kabashi Q, Goga I, Berisha B (2006). Evidence for the presence of bluetongue virus in Kosovo between 2001 and 2004. Vet Rec.

[pbio-0060210-b009] Singer RS, MacLachlan NJ, Carpenter TE (2001). Maximal predicted duration of viraemia in bluetongue virus-infected cattle. J Vet Diagn Invest.

[pbio-0060210-b010] Nasci RS, Savage HM, White DJ, Miller JR, Cropp BC (2001). West Nile virus in overwintering Culex mosquitoes, New York City, 2000. Emerg Infect Dis.

[pbio-0060210-b011] Lindsay MDA, Broom AK, Wright AE, Johansen CA, MacKenzie JS (1993). Ross River virus isolations from mosquitoes in arid regions of Western Australia: Implications of vertical transmission as a means of persistence of the virus. Am J Trop Med Hyg.

[pbio-0060210-b012] Goddard LB, Roth AE, Reisen WK, Scott TW (2003). Vertical transmission of West Nile virus by three California Culex (Diptera: Culicidae) species. J Med Entomol.

[pbio-0060210-b013] Reisen WK, Fang Y, Lothrop HD, Martinez VM, Wilson J (2006). Overwintering of West Nile virus in southern California. J Med Entomol.

[pbio-0060210-b014] Tsai TF (2006). Congenital arboviral infections: Something new, something old. Pediatrics.

[pbio-0060210-b015] Aaskov JG, Davies CEA, Tucker M, Dalglish D (1981). Effect on mice of infection during pregnancy with three Australian arboviruses. Am J Trop Med Hyg.

[pbio-0060210-b016] Kettle DS (1962). The bionomics and control of Culicoides and Leptoconops (Diptera, Ceratopogonidae=Heleidae). Annu Rev Entomol.

[pbio-0060210-b017] Mellor PS (1990). The replication of bluetongue virus in Culicoides vectors. Curr Top Microbiol Immunol.

[pbio-0060210-b018] White DM, Wilson WC, Blair CD, Beaty BJ (2005). Studies on overwintering of bluetongue viruses in insects. J Gen Virol.

[pbio-0060210-b019] Nunamaker RA, Sieburth PJ, Dean VC, Wigington JG, Nunamaker CE (1990). Absence of transovarial transmission of bluetongue virus in Culicoides variipennis: Immunogold labelling of bluetongue virtus antigen in developing oocytes from Culicoides variipennis (Coquillett). Comp Biochem Physiol A.

[pbio-0060210-b020] Mellor PS, Boorman J, Baylis M (2000). Culicoides biting midges: Their role as arbovirus vectors. Annu Rev Entomol.

[pbio-0060210-b021] Lysyk TJ, Danyk T (2007). Effect of temperature on life history parameters of adult Culicoides sonorensis (Diptera: Ceratopogonidae) in relation to geographic origin and vectorial capacity for bluetongue virus. J Med Entomol.

[pbio-0060210-b022] Losson B, Mignon B, Paternostre J, Madder M, De Deken R (2007). Biting midges overwintering in Belgium. Vet Rec.

[pbio-0060210-b023] European Food Safety Authority (2007). Epidemiological analysis of the 2006 bluetongue virus serotype 8 epidemic in north-western Europe. http://www.efsa.europa.eu/EFSA/efsa_locale-1178620753812_Bluetongue.htm.

[pbio-0060210-b024] Hourrigan J, Klingsporn A (1975). Bluetongue: The disease in cattle. Aust Vet J.

[pbio-0060210-b025] Luedke AJ, Jones RH, Walton TE (1977). Overwintering mechanism for bluetongue virus: Biological recovery of latent virus from a bovine by bites of Culicoides variipennis. Am J Trop Med Hyg.

[pbio-0060210-b026] Takamatsu H, Mellor PS, Mertens PPC, Kirkham PA, Burroughs JN (2003). A possible overwintering mechanism for bluetongue virus in the absence of the insect vector. J Gen Virol.

[pbio-0060210-b027] Gibbs EPJ, Lawman MJP, Herniman KAJ (1979). Preliminary observations on transplacental infection of bluetongue virus in sheep—A possible overwintering mechanism. Res Vet Sci.

[pbio-0060210-b028] Gildernew M (2008). Ministerial Statement: Update on bluetongue. Official report (Hansard) of the Northern Ireland Assembly.

[pbio-0060210-b029] De Clerq K, Vandenbussche F, Vandemeulebroucke E, Vanbinst T, De Leeuw I (2008). Transplacental bluetongue infection in cattle. Vet Rec.

[pbio-0060210-b030] Wouda W, Roumen MPHM, Peperkamp NHMT, Vos JH, van Garderen E (2008). Hydranencephaly in calves following the bluetongue serotype 8 epidemic in the Netherlands. Vet Rec.

[pbio-0060210-b031] Murray JO, Trainer DO (1970). Bluetongue virus in North American Elk. J Wildl Dis.

[pbio-0060210-b032] Sohn R, Yuill T (1991). Bluetongue and epizootic haemorrhagic disease in wild ruminants. Bull Soc Vector Ecol.

[pbio-0060210-b033] Linden A, Mousset B, Hanrez D, Vandenbussche F, Vandemeulebroucke E (2008). Bluetongue virus antibodies in wild red deer in southern Belgium. Vet Rec.

[pbio-0060210-b034] Swellengrebel N, de Buck A (1938). Malaria in the Netherlands.

[pbio-0060210-b035] Stott JL, Osburn BI, Alexander L (1985). Ornithodorus coriaceus (Pajaroello tick) as a vector of bluetongue virus. Am J Vet Res.

[pbio-0060210-b036] Luedke AJ, Jochim MM, Bowne JG (1965). Preliminary bluetongue transmission with the sheep ked Melophagus ovinus (L.). Can J Comp Med Vet Sci.

[pbio-0060210-b037] Alexander KA, MacLachlan NJ, Kat PW, House C, O'Brien SJ (1994). Evidence of natural bluetongue infection among African carnivores. Am J Trop Med Hyg.

[pbio-0060210-b038] Akita G, Ianconescu M, MacLachlan NJ, Osburn BI (994). Bluetongue disease in dogs associated with contaminated vaccine. Vet Rec.

[pbio-0060210-b039] Meiswinkel R, Baldet T, De Deken R, Delécolle J-C, Takken W (2008). The 2006 outbreak of bluetongue in northern Europe-the entomological perspective. Prev Vet Med.

[pbio-0060210-b040] Belgium Food Agency (FAVV/AFSCA) (2008). Réapparition de la fièvre catarrhale des ovins chez les ruminants en Belgique. http://www.favv-afsca.be/home/press/_documents/2008-07-29_blauwtongvirus_fr.pdf.

[pbio-0060210-b041] International Society for Infectious Diseases (2008). Bluetongue - Europe (36): BTV-8, Germany, ovine, first case 2008. http://www.promedmail.org/pls/otn/f?p=2400:1202:3895934457993676::NO::F2400_P1202_CHECK_DISPLAY,F2400_P1202_PUB_MAIL_ID:X,73223.

